# Finite element study on the micromechanics of cement-augmented proximal femoral nail anti-rotation (PFNA) for intertrochanteric fracture treatment

**DOI:** 10.1038/s41598-024-61122-2

**Published:** 2024-05-06

**Authors:** Yurui Liu, Liqin Zheng, Shaobin Li, Zhengze Zhang, Ziling Lin, Wuhua Ma

**Affiliations:** 1 Department of Anesthesiology, Beijing Stomatological Hospital, School of Stomatology, Capital Medical University, Beijing, China; 2 Department of Orthopedics, The First Affiliated Hospital of Guangzhou University of Chinese Medicine, Guangzhou, China; 3 The First Clinical Medical College, Guangzhou University of Chinese Medicine, Guangzhou, China; 4 Department of Anesthesiology, The First Affiliated Hospital of Guangzhou University of Chinese Medicine, Guangzhou, China

**Keywords:** Intertrochanteric fracture, Proximal femoral nail anti-rotation, Bone cement, Trabecular bone, Micromechanics, Finite element method, Trauma, Orthopaedics

## Abstract

Blade cut-out is a common complication when using proximal femoral nail anti-rotation (PFNA) for the treatment of intertrochanteric fractures. Although cement augmentation has been introduced to overcome the cut-out effect, the micromechanics of this approach remain to be clarified. While previous studies have developed finite element (FE) models based on lab-prepared or cadaveric samples to study the cement-trabeculae interface, their demanding nature and inherent disadvantages limit their application. The aim of this study was to develop a novel 'one-step forming' method for creating a cement-trabeculae interface FE model to investigate its micromechanics in relation to PFNA with cement augmentation. A human femoral head was scanned using micro-computed tomography, and four volume of interest (VOI) trabeculae were segmented. The VOI trabeculae were enclosed within a box to represent the encapsulated region of bone cement using ANSYS software. Tetrahedral meshing was performed with Hypermesh software based on Boolean operation. Finally, four cement-trabeculae interface FE models comprising four interdigitated depths and five FE models comprising different volume fraction were established after element removal. The effects of friction contact, frictionless contact, and bond contact properties between the bone and cement were identified. The maximum micromotion and stress in the interdigitated and loading bones were quantified and compared between the pre- and post-augmentation situations. The differences in micromotion and stress with the three contact methods were minimal. Micromotion and stress decreased as the interdigitation depth increased. Stress in the proximal interdigitated bone showed a correlation with the bone volume fraction (R^2^ = 0.70); both micromotion (R^2^ = 0.61) and stress (R^2^ = 0.93) at the most proximal loading region exhibited a similar correlation tendency. When comparing the post- and pre-augmentation situations, micromotion reduction in the interdigitated bone was more effective than stress reduction, particularly near the cement border. The cementation resulted in a significant reduction in micromotion within the loading bone, while the decrease in stress was minimal. Noticeable gradients of displacement and stress reduction can be observed in models with lower bone volume fraction (BV/TV). In summary, cement augmentation is more effective at reducing micromotion rather than stress. Furthermore, the reinforcing impact of bone cement is particularly prominent in cases with a low BV/TV. The utilization of bone cement may contribute to the stabilization of trabecular bone and PFNA primarily by constraining micromotion and partially shielding stress.

## Introduction

**Figure 1:**
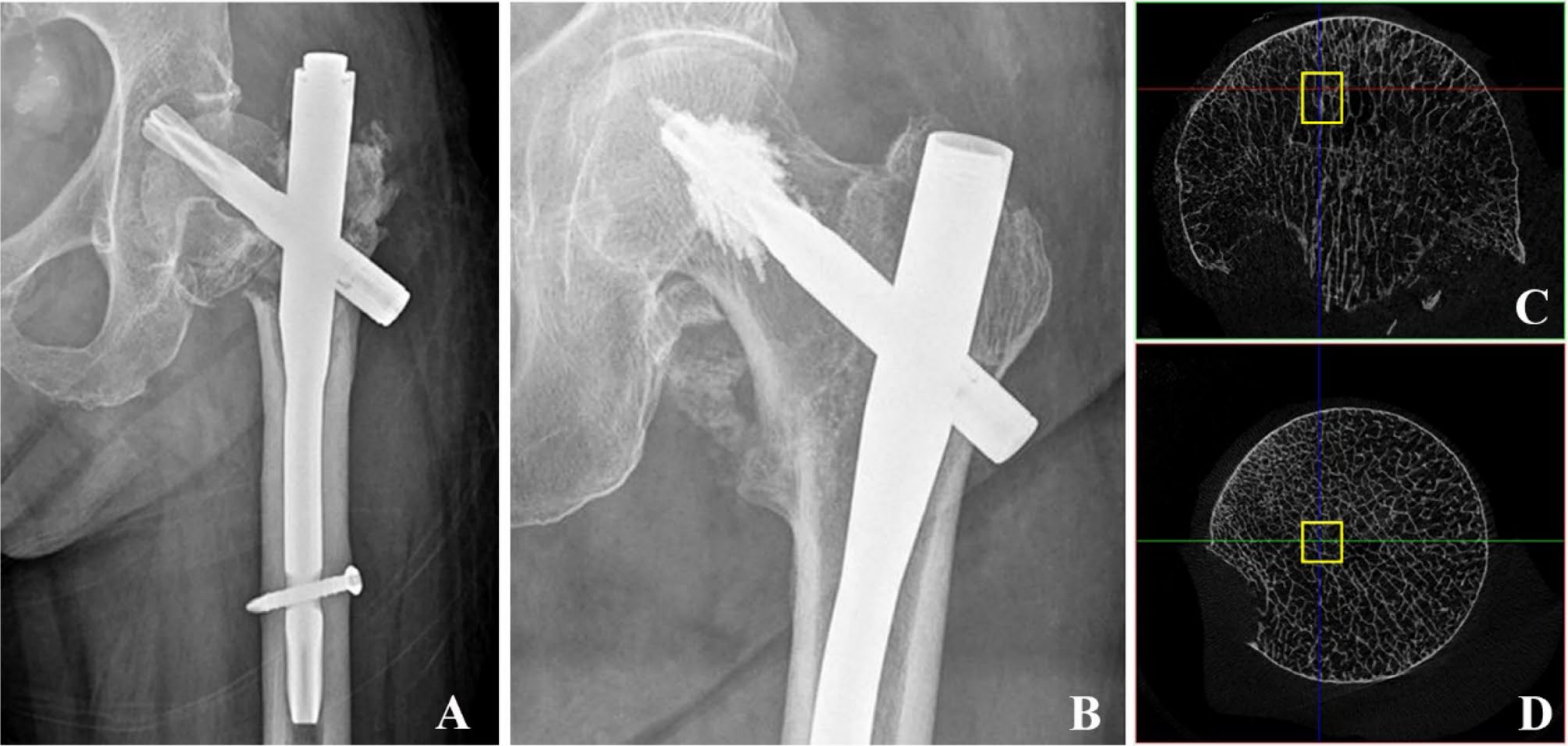
(**A**) Radiographic image of a blade cut-out extending from the femoral head into the acetabulum^29^. (**B**) Radiographic image of a completely recovered intertrochanteric fracture after cement-augmented PFNA fixation^8^. Figures were reproduced with permission from Elsevier. (**C**) and (**D**) VOI of trabecular bone segmented from the femoral head. The axis of VOI is aligned with the main compressive trabecular direction.

The initial stability of bone cement is of interest in many orthopaedic procedures, including treatments for proximal femur and vertebral fragility fractures as well as total hip/knee replacements. This is because bone cement enhances the interface stability between the implant and osteoporotic trabecular tissue, thereby reducing the risk of implant loosening and subsequent revision operations^1–5^. Proximal femoral nail anti-rotation (PFNA) has become the preferred intramedullary fixation technique for treating intertrochanteric fractures. However, as the number of cases increases, so does the incidence of fixation-related complications such as blade cut-out, which is reported to occur in approximately 2–15% of cases^6,7^. (Fig. 1A).

Cement-augmented PFNA has been proposed for over a decade since its first clinical application^8^ (Fig. 1B). The biomechanical benefits of bone cement augmentation, such as prevention of cut-out, rotation of the femoral head, and resistance to fatigue, have been demonstrated, as exemplified by PFNA, which employs a helical blade and later-injected cement volume^9–11^. In a previous study simulating cement-augmented PFNA for the treatment of stable and unstable intertrochanteric fractures, we found that bone cement effectively enhanced the stability of PFNA and reduced the risk of cut-out by dispersing and transferring stress from the blade tip to both the nail and femoral shaft^12^. Clinically, even with a higher tip apex distance or calcar-referenced tip apex distance, augmenting the PFNA blade has the advantage of preventing reoperations by strengthening the osteosynthetic construct to resist symptomatic implant migration^13,14^. Furthermore, cement-augmented PFNA significantly increases the post-operative loading rate, which describes the force with which the foot strikes the ground during walking and combines two variables: mobility and pain^15^. Besides, cement augmentation reportedly carries a low risk of negative biological side effects, including pressure/temperature-induced avascular necrosis, because limited cement amounts are injected at low pressure^16–18^. The safety of the procedure has been demonstrated in terms of preventing cement leakage into the joint, minimizing mechanical failures like cut-out, reducing mortality rates, and decreasing post-operative complications^19^.

Although the technique of cement-augmented PFNA has been successfully introduced in macro-biomechanical tests and clinical studies to increase the stability of osteoporotic femoral heads, the mechanism of cement augmentation at a smaller trabecular dimension remains unclear. This can be understood by simulating the interaction between trabeculae and bone cement using the finite element method (FEM). The FEM is a computer-based method used to predict how an object will respond to specific physical conditions, such as loading or heat transfer, in the real world. This is achieved by dividing the object into finite discrete elements, each with its own mathematical equation that describes its mechanical behaviour. The FEM has been extensively used to study the mechanical behaviour of trabecular bone under various loading conditions and examine the distribution of stresses, displacements, or strains within the bone structure.

Previously, researchers utilized the FEM to observe cement-trabeculae interactions and quantify the strain shielding of cement at the trabecular scale, using lab-prepared or post-mortem-retrieved samples from the cement-trabeculae interface in total knee arthroplasty^20–25^. However, these interface samples introduce inherent limitations or errors into the analysis. First, the demanding nature of modelling cement–bone interface limits the number of specimens that can be tested, validated, and modelled. Second, micro- and macro-gaps between the cement and trabeculae have frequently been discovered^20,26,27^, and there is room for improvement in terms of accuracy in mechanical analysis. Third, in order to investigate pre- and postoperative differentiation in osteolysis accurately, it is necessary to manually fill the cavity that are time-consuming^20^. Fourth, when utilizing micro-computed tomography (micro-CT) scanning for investigation purposes, there is a significant overlap between the global thresholds of trabeculae and bone cement^26,28^; investigators have to manually segment the cement layer and trabeculae, which could potentially introduce additional artificial interference by unintentionally altering the original structure of trabeculae. Therefore, a user-friendly, universal, and accurate model of cement-trabeculae needs to be developed for further investigation.

In this study, we developed a finite element (FE) model of the cement-trabeculae interface without using lab-prepared or post-mortem retrieved specimens. The FE models were theoretically free from the aforementioned disadvantages and were used to determine: (1) how micromotion and stress vary within the interdigitated trabeculae at the cement-trabeculae interface; (2) whether the interdigitated trabecular bone is constrained in terms of micromotion or shielded in terms of stress compared to the bone distal to the interface; and (3) what extent of micromotion constraint or stress shielding exists at the cement–bone interface compared to non-augmented bone.

## Methods

### Sample preparation and micro-CT scanning

We recruited an 83-year-old man who underwent hip hemiarthroplasty for a femoral neck fracture caused by a sideways fall. The subject provided informed consent and signed it prior to participating in this study. The study was approved by the institutional ethics committee of the First Affiliated Hospital of Guangzhou University of Chinese Medicine (approval number: JY 2019-101). All experiments were performed in accordance with the Declaration of Helsinki.

The right femoral head was extracted during hip hemiarthroplasty, fixed with 4% paraformaldehyde for 72 h, and then scanned using micro-CT (SkyScan 1276; Bruker, USA. https://blue-scientific.com/bruker-micro-ct-software/) at 42 μm resolution. The X-ray source voltage of micro-CT was set to 100 kV, and the current was set to 200 μA. A rotation step as low as 0.8° was selected to obtain the finest resolution. Based on the micro-CT images, 8-bit bitmaps of every cross-section along the femoral neck axis were reconstructed using the bundled Nrecon software, version 1.7.1 (Bruker, USA). Subsequently, the Dataviewer 1.5.6 (Bruker, USA) software was employed to orient the cross-sectional images perpendicular to the main compressive trabecular direction in the femoral head. Finally, a global threshold of 45–255 was chosen to segment the trabecular bone using the CTAn 1.16.8 (Bruker, USA) software. Four quadrangular prism trabecular samples with dimensions of 3 × 3 × 4 mm were randomly obtained from the domain of the femoral head as the volume of interest (VOI). The VOI axis was aligned with the main compressive trabecular direction (Fig. 1C, D). The VOI trabecular quadrangular prisms were saved in a ‘stl’ file format for subsequent FE model generation, and their bone volume fraction (BV/TV) was recorded.

### Pre- and post-augmentation FE model generation

**Figure 2:**
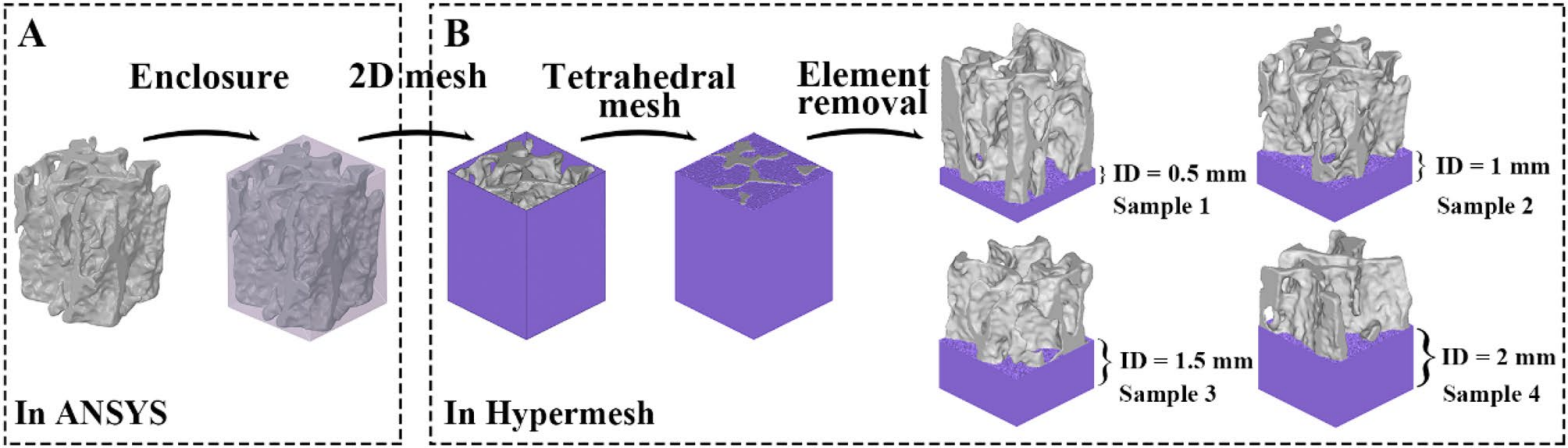
Steps taken for the development of cement-trabeculae interface FE model. (**A**) Enclosure procedure was performed in the SpaceClaim module of the ANSYS 19.0 software. (**B**) The 2D surface and 3D tetrahedral meshing were utilized to create the cement volume region, followed by the removal of elements to produce a cement interdigitated model comprising four interdigitated depths (ID).

To eliminate disconnected elements in the VOI trabeculae, which can result in modeling and calculation failures, optimization techniques such as smoothing, mesh reduction, and remeshing were implemented using Geomagic studio 2013 (GEOMAGIC, USA. https://oqton.com/geomagic-wrap/). The optimizations were carefully performed to reduce noise while preserving the original trabecular structure as much as possible. The refined trabecular samples were saved as 'stl' files. Subsequently, the trabecular sample was wrapped in an enclosure box with dimensions of 3.01 × 3.01 × 4.01 mm, representing the original encapsulated region of bone cement, using the SpaceClaim module of ANSYS software, version 19.0 (ANSYS, USA. https://www.ansys.com/). The enclosure box and trabecular sample were converted to solid components and saved as ‘stl’ files (Fig. 2A). Two-dimensional (2D) surface and three-dimensional (3D) tetrahedral meshing were executed sequentially using Hypermesh 14.0 (Altair, USA. https://altair.com/hypermesh). Specifically, the cement region (enclosure box) was tetrahedrally meshed by the Boolean logic operation while keeping the 2D trabecular surface active during the meshing process. After tetrahedrally meshing the trabecular bone volume, a portion of volumetric cement elements were removed from the proximal region to establish a FE model of cement-trabeculae interface with four interdigitated depths (IDs), including 0.5, 1.0, 1.5, and 2 mm (Fig. 2B). The elements were all kept as first-order elements. To assess the changes in micromotion and stress induced by cementation, the blank trabecular samples were duplicated and saved as pre-augmentation specimens following optimization management but prior to the enclosure procedure. Further, five additional FE model of cement-trabeculae interface with different BV/TV were also established to assess the micromotion and stress changes.

**Figure 3:**
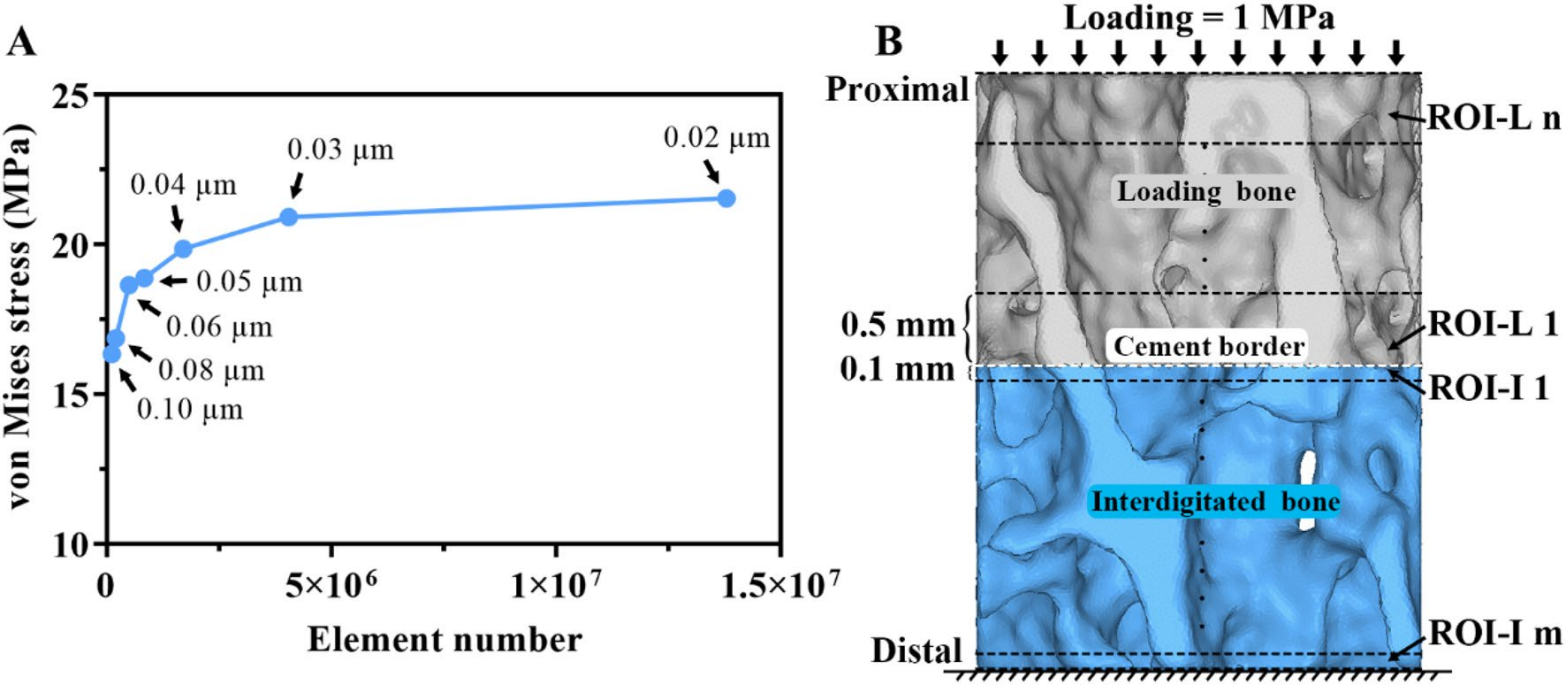
Convergence test and the ROIs within the FE model. (**A**) The element size of 0.04 mm was sufficient for convergence. (**B**) The interdigitated bone was divided into serial ROIs at intervals of 0.1 mm thickness (denoted as ROI-I), while the loading bone is divided into segments of 0.5 mm thickness (denoted as ROI-L). The counting number (m) of the ROI-I depend on the interdigitated depth, while counting number (n) of the ROI-L depend on loading bone height in each model. The cement border (white dashed line) has zero interdigitated depth and the most distal region has the maximum. Loading bone is the bone present above the cement border.

Due to variations in the IDs and trabecular structure, the total number of elements in the cement-trabeculae interface (post-augmentation) models ranged from 2.29 to 4.27 million with 0.42 to 0.75 million nodes. The element density was kept sufficiently high for an accurate representation of the trabecular bone microstructure. The average element size for all models was set to 0.04 mm, because the convergence test suggested that a size of 0.04 mm was sufficient^22^ **(**Fig. 3A).

### Boundary condition and loading

Boundary condition and loading setting were operated in Hypermesh 14.0 software. The bottom surface of cement-trabeculae interface model was fully constrained in all degrees of freedom while while a uniaxial static load was applied to the top surface of trabecular bone. Linear elastic and isotropic material properties were assumed in the FE models. The Young’s moduli of the cement and trabecular elements were set at 3 and 14 GPa^28^, respectively. A Poisson’s ratio of 0.3 was applied for both cement and bone. Friction, frictionless, and bond contact were established to compare the effect of the interfacial properties between the cement and bone, where friction contact with a coefficient of 0.1, 0.3, 0.5, 0.7 and frictionless contact were modelled as double-sided segment-to-segment contact without friction^26,30^. All FE models, including pre- and post-augmentation models, were subjected to a uniaxial static compression load of 1 MPa, which is equivalent to one body weight^20^. All the simulations were performed on the Optistruct module of the Hypermesh 14.0 software. The micromotion and von Mises stress of the trabeculae were exported and saved after calculation using Hyperview module in Hypermesh 14.0.

### Definition of regions of interest (ROI) in the interdigitated bone

To enable the post-processing of micromotion and stress, the interdigitated bone was divided into serial ROIs with equal thickness of 0.1 mm (denoted as ROI-I), while the loading bone was divided into segments with a thickness of 0.5 mm (denoted as ROI-L). The ROIs were numbered from the cement border to the double distal end** (**Fig. 3B) and defined in both post-augmentation and pre-augmentation models to facilitate comparison. The maximum micromotion and von Mises stress were recorded from each ROI, and the values for ROI-I and ROI-L were respectively defined as functions of their corresponding ID and loading height. Specifically, the cut-off values for the upper 95th percentile (i.e. top 5%) of micromotion and stress in each ROI were defined to represent the maximum levels^31,32^. In more detail, all micromotion and von Mises data points were exported from each ROI and sorted in descending order. The maximum value of the top 5% were then extracted for further statistical analysis. The micromotion and stress of the interdigitated bone were also compared with those in the loading bone.

## Results

### Effect of contact method

**Figure 4:**
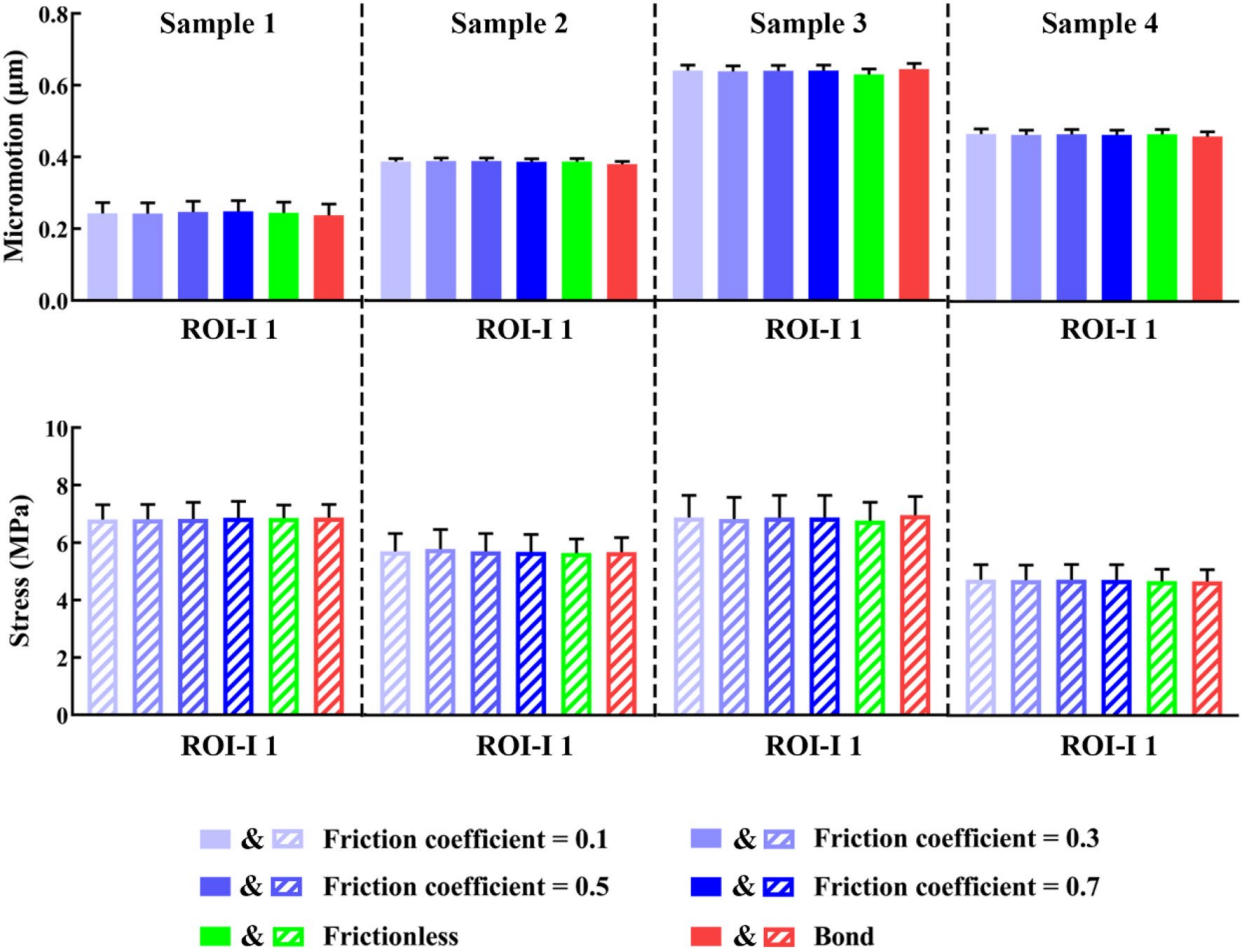
Comparison among contact methods/coefficient in terms of maximum micromotion and stress of each cement-trabeculae sample. No significant differences were found among different contact methods/coefficient (*P* > 0.05, as measured by one-way ANOVA).

The ROI-I 1, which refers to the interdigitated bone closest to the cement border, was utilized as a reference for comparison among all samples. The impact of various contact methods, including friction coefficients of 0.1, 0.3, 0.5, and 0.7, frictionless contact, and bonding at the interface between cement and trabeculae on maximum micromotion and stress is illustrated in Fig. 4. From sample 1 (ID = 0.5 mm) to sample 4 (ID = 2 mm), the maximum difference in micromotion due to the three contact methods and coefficient was found to be 4.27%, 2.40%, 2.34%, and 1.62% respectively, while the maximum difference in stress was found to be 1.05%, 2.54%, 2.78%, and 1.34% respectively. The findings suggest that the contact method employed did not have a significant impact on the mechanical behaviour of the cement–bone interface. Consequently, we opted to utilize a frictionless contact method for subsequent analyses, aligning with previous studies^21,23,26^.

### Distribution of micromotion in interdigitated trabeculae

**Figure 5:**
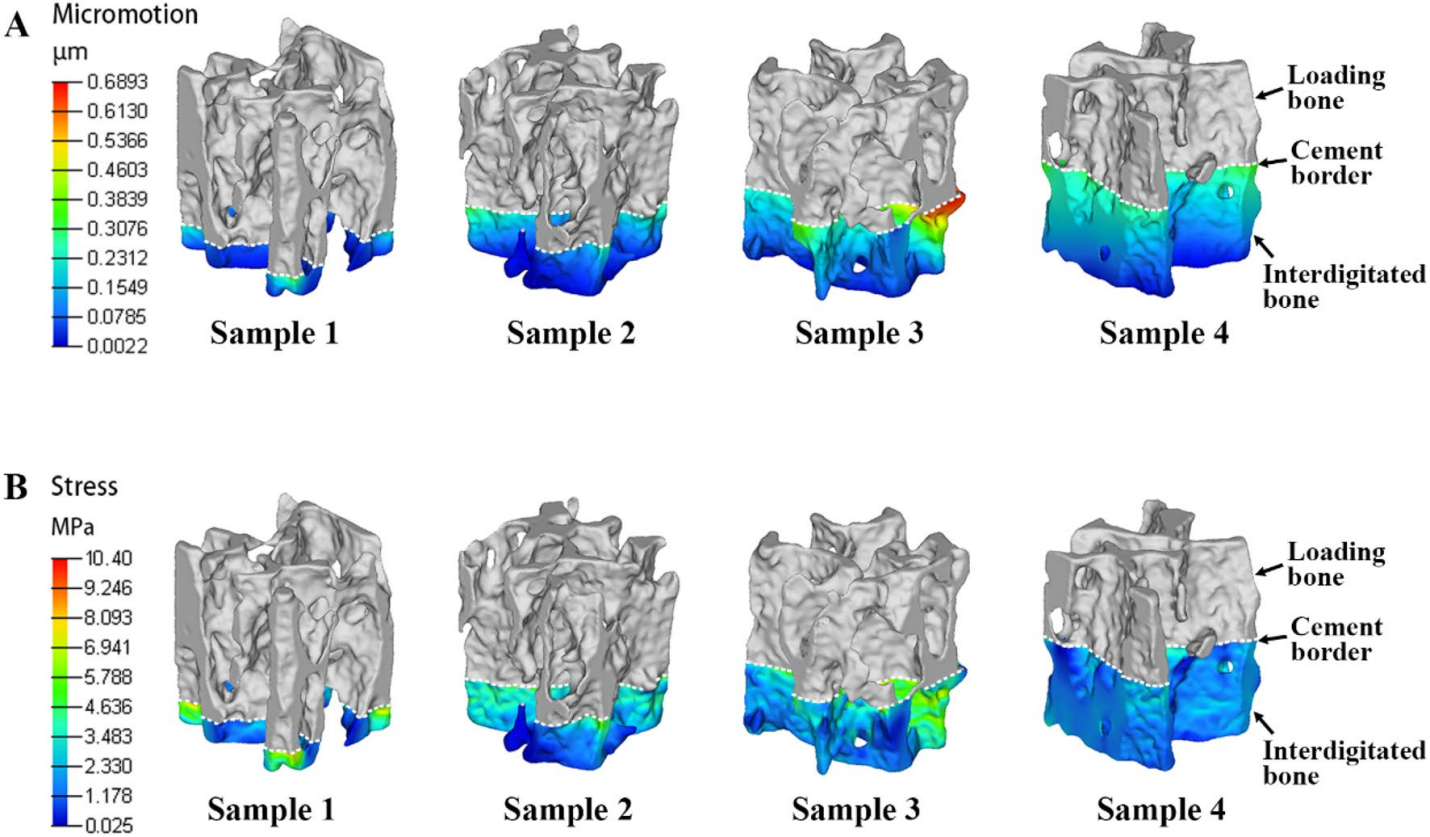
Variation of micromotion and stress in the interdigitated bone. Both micromotion (**A**) and stress (**B**) of the interdigitated bone exhibit an increasing trend closer to the cement border. The contour plot illustrates the interdigitated bone without the cement layer for clarity.

**Figure 6:**
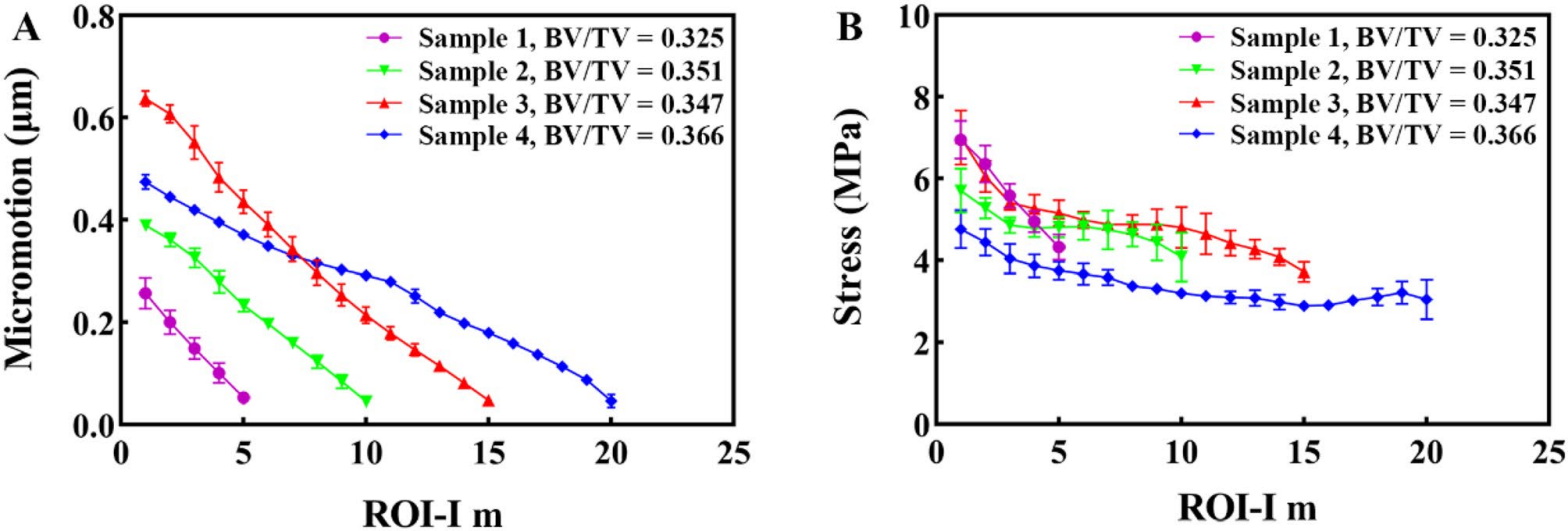
Micromotion and stress as a function of interdigitated depth. (**A**) Micromotion and (**B**) stress data obtained from the FE models are presented as maximum plots for ROI-I, representing their functions with respect to the interdigitation depth.

Trabecular micromotion decreased from the proximal to the distal interdigitated bone, as shown in the FE contour plot** (**Fig. 5A). The micromotion in each ROI-I, as a function of the ID, showed that deeper regions within the cement layer had lower micromotion (Fig. 6A). In ROI-I 1, variations in micromotion were observed among four cement-trabeculae models. However, these variations did not appear to correlate with changes in BV/TV (R^2^ = 0.02); samples with lower BV/TV values displayed less micromotion at the contact interface. The micromotion in each ROI was assessed using one-way ANOVA and demonstrated significant differences compared to the other regions (*P* < 0.05). This observation was consistent across all samples.

### Distribution of stress in interdigitated trabeculae

The stress in the interdigitated trabeculae exhibited a similar decreasing trend from the proximal to the distal region for each sample. The stress patterns of all four samples were compared, and a decreasing trend was observed with increasing ID** (**Fig. 5B). Within each sample, it was expected that the interdigitated bone stress would decrease as the cement-mantle thickness increased (Fig. 6B). In contrast to the micromotion trend, when observing the stress at ROI-I 1, there was a tendency for stress to increase with decreasing BV/TV (R^2^ = 0.70), suggesting that bone quantity may impact load transfer in this region.

### Interdigitated and loading bone micromotion

**Figure 7:**
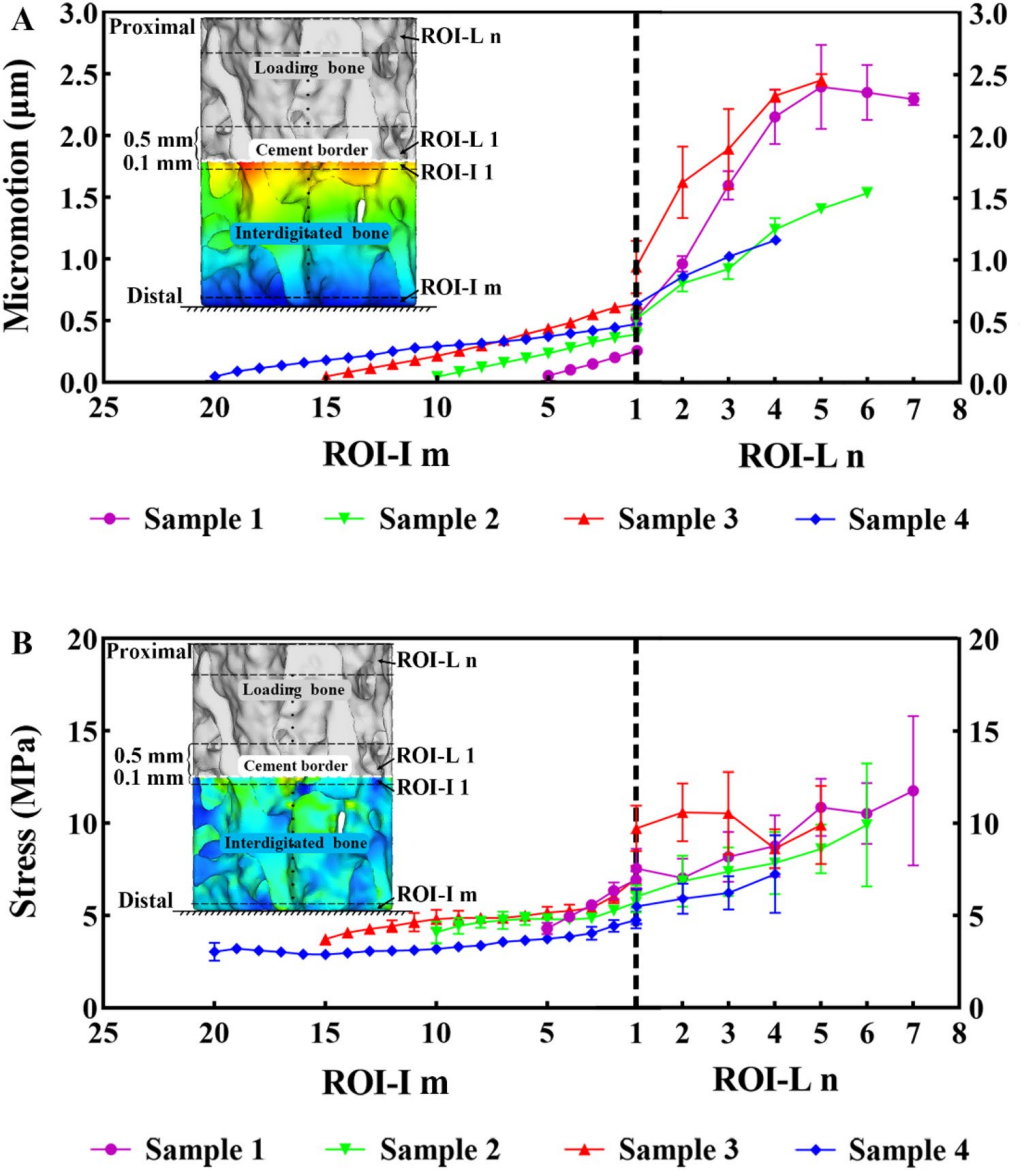
Comparison of micromotion and stress between the interdigitated and loading bone. The (**A**) micromotion and (**B**) stress in the interdigitated bone are significantly lower than those in the loading bone.

The micromotion data from all regions of the interdigitated bone indicated that the interdigitated bone was micromotion-constrained compared with the loading bone. The constraint effect increased with the ID, with a more evident micromotion difference observed in the distal ROI-I **(**Fig. 7A). The distal loading bone (near the cement border) was also micromotion-constrained compared with the proximal loading bone. A thinner cement mantle corresponded to a higher micromotion gradient in the loading bone. Additionally, there was a strong correlation between the micromotion of the most proximal ROI-L n (R^2^ = 0.61) and the BV/TV in comparison to ROI-L 1 (R^2^ = 0.03).

### Interdigitated and loading bone stresses

In accordance with the variation of micromotion, the stress data obtained from all interdigitated bone regions exhibited a noticeable trend of stress shielding in comparison to the loading bone. Moreover, there was a positive correlation between the degree of stress shielding and ID **(**Fig. 7B). The stress variation between the interdigitated and loading bones exhibited a lower magnitude, as indicated by the slope of the curves, compared to that of micromotion variation. The distal loading bone (near the cement border) was also stress-shielded compared with the proximal loading bone. There was a strong correlation between the stress of the most proximal ROI-L n (R^2^ = 0.93) and the BV/TV in comparison to ROI-L 1 (R^2^ = 0.22).

### Micromotion and stress variations between pre- and post-augmentation models

**Figure 8:**
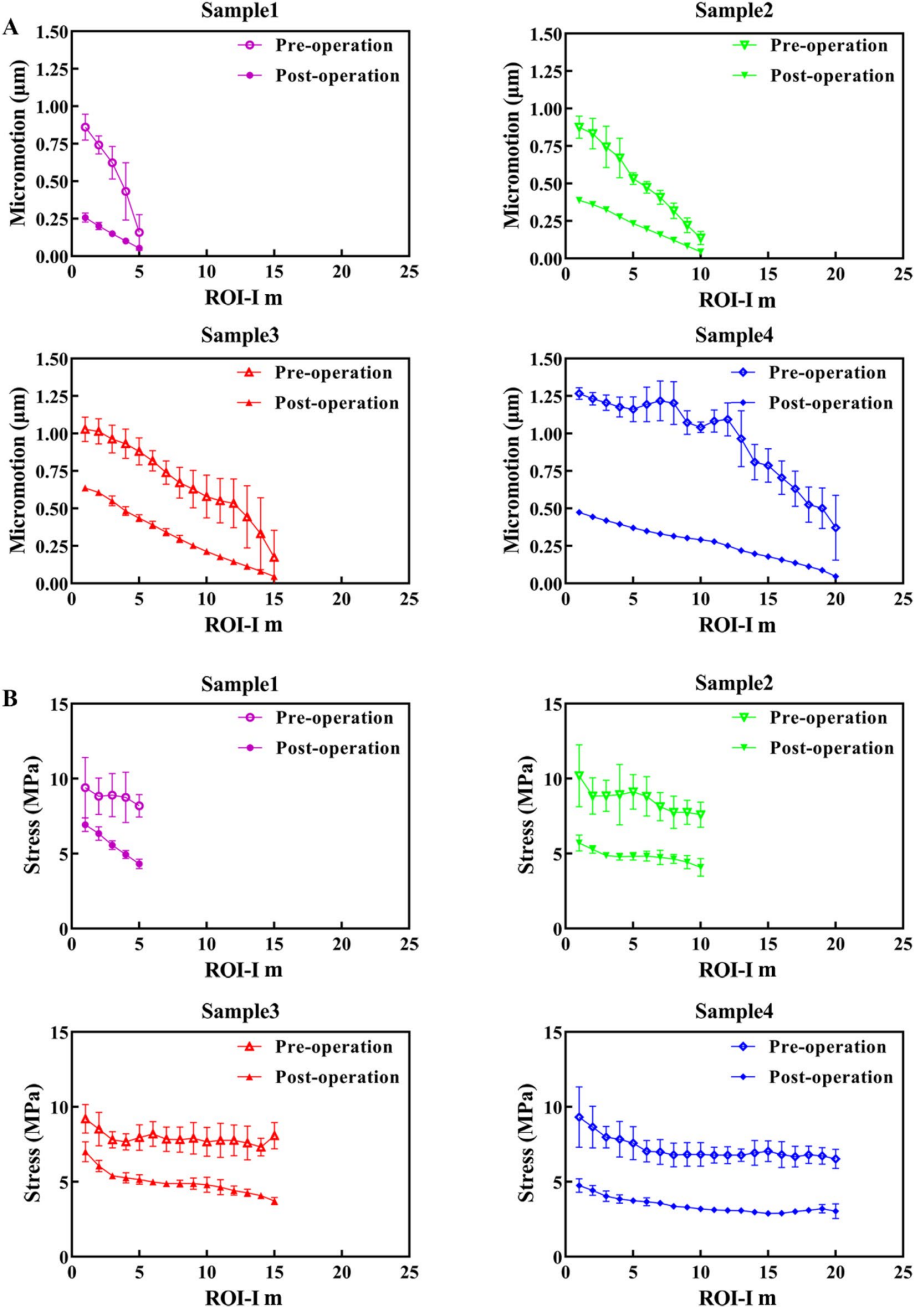
Micromotion and stress in the interdigitated bone before and after cement augmentation. The interdigitated bone is (**A**) micromotion-constrained and (**B**) stress-shielded due to the presence of the cement layer.

Figure 8 shows the micromotion and stress in the interdigitated bone in the pre- and post-augmentation situations. The micromotion and stress of the proximal ROI were higher than those of the more distal ROI, indicating that there was micromotion constraint and stress shielding in the most distal region. The reduction of micromotion in the interdigitated bone is superior to stress reduction, particularly in the regions adjacent to the cement border, exhibiting maximum percentage decreases of 70.1%, 55.6%, 38.1%, and 62.5% for the four samples, respectively.

**Figure 9:**
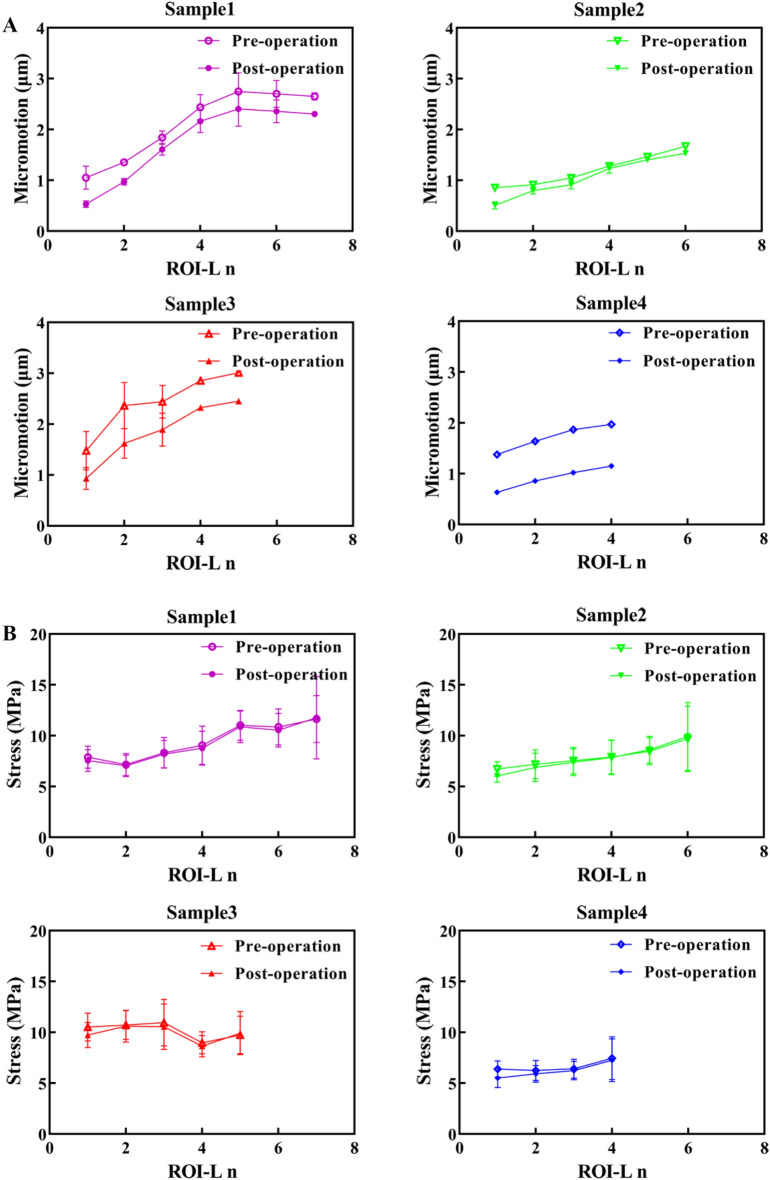
Micromotion (**A**) and stress (**B**) in the loading bone before and after cement augmentation. Loading bone may be micromotion-constrained while free from stress shielding after cementation.

The micromotion and stress in the loading bone before and after cementation are illustrated in Fig. 9. Cementation resulted in a significant reduction in micromotion within the loading bone, while the decrease in stress was minimal. This asymmetrical reduction suggests that the strengthening effect of bone cement on the loading bone may rely on constraining micromotion.

### Effect of BV/TV on micromotion and stress variations in interdigitated bone

**Figure 10:**
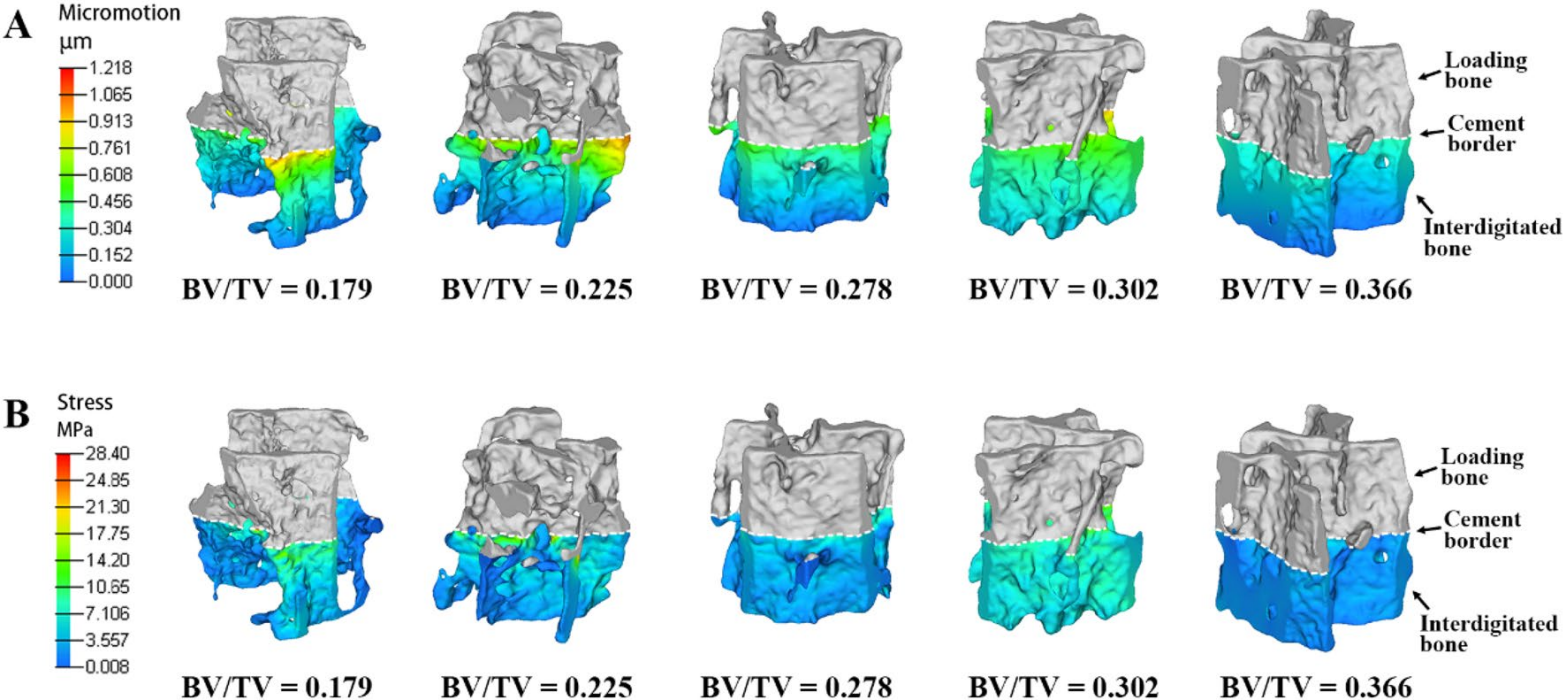
Distribution of micromotion and stress in interdigitated bone varies among different BV/TV samples. The (**A**) micromotion and (**B**) stresses of the interdigitated bone exhibit an increasing trend as BV/TV decreases. The contour plot illustrates the interdigitated bone without a cement layer for clarity.

**Figure 11:**
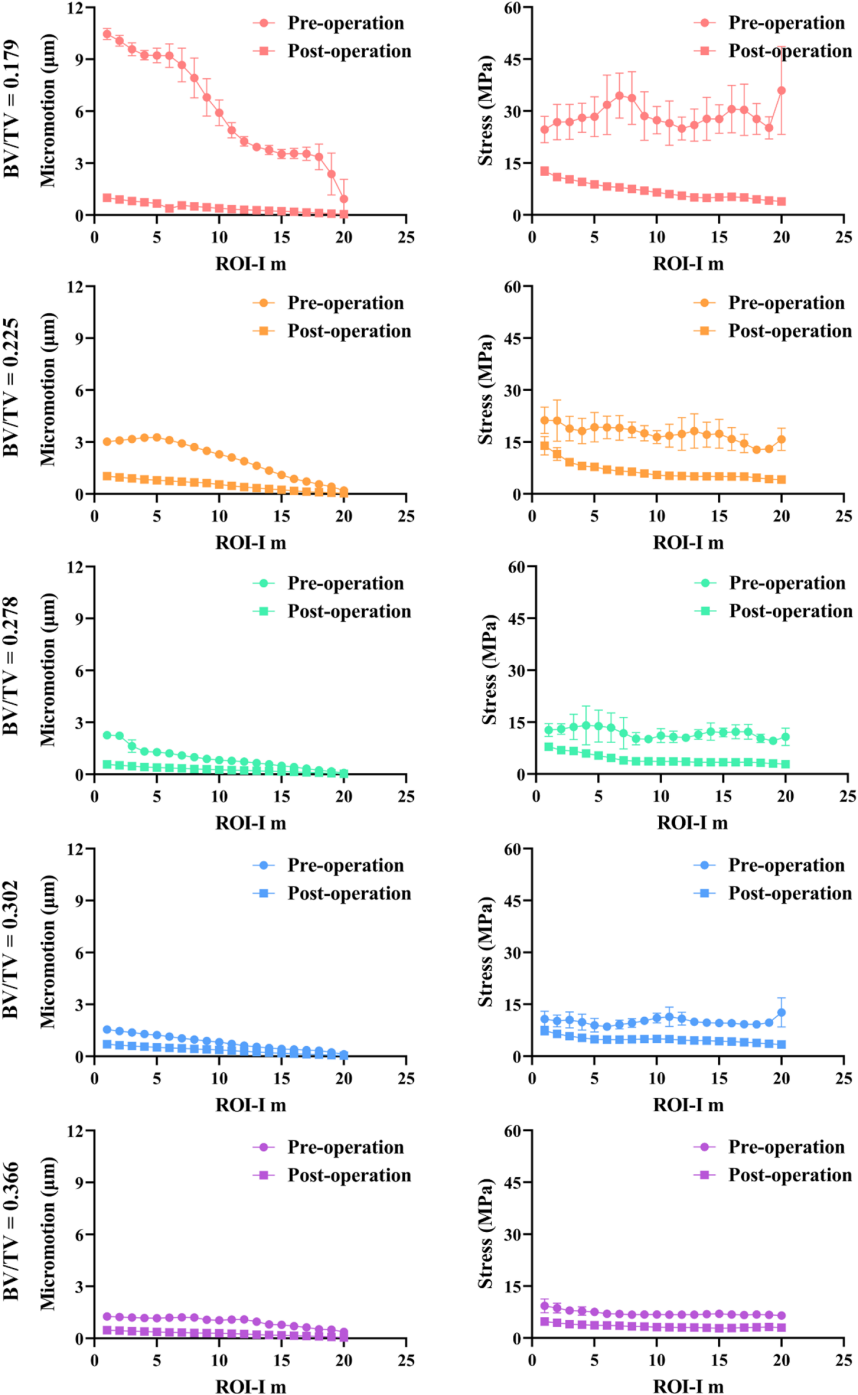
Micromotion and stress in the interdigitated bone before and after cement augmentation indicate that trabeculae with lower BV/TV are more susceptible to bone cement augmentation.

The present study developed additional cement-trabeculae interface models with varying BV/TV values (0.179, 0.225, 0.278, 0.302 and 0.366) to investigate the micromotion and stress variations in interdigitated bone. With lower BV/TV, trabeculae are more susceptible to deformation and stress concentration, particularly those near the cement border **(**Fig. 10). The presence of micromotion and stress in interdigitated bone both before and after cement augmentation indicates that trabeculae with a lower BV/TV exhibit greater sensitivity towards this procedure, as evidenced by a significant reduction in micromotion and stress levels **(**Fig. 11). These findings suggest that individuals with lower bone mass may benefit from more effective mechanical support provided by bone cement augmentation.

## Discussion

This study introduces user-friendly, universal, and accurate finite element (FE) models to investigate the micromechanics of the cement-trabeculae interface. The aim is to microscopically elucidate the mechanism of treating intertrochanteric fractures with cement-augmented PFNA. Samples with varying cement IDs and BV/TV were used to study the effect of bone morphology on micromotion and stress. "One-step forming" refers to automatically filling cement elements without a lab-prepared or cadaveric cement-bone sample. Our study revealed that both micromotion and stress decreased when moving away from the cement implant, with the highest micromotion and stress observed at the cement border due to cement interdigitation. Although there is a lack of experimental or computational verification for the femoral head cement-trabeculae interface, our results are consistent with previous findings from lab-prepared specimens^33^ and FE simulations^20,28^ on proximal tibial trabeculae, where similar magnitudes of micromotion were measured. In clinical practice, little attention has been given to the role of the implant–cement interface due to its strength and infrequent fixation failure. Instead, failures such as secondary micro fracture of trabecuale, cement leakage, and osteolysis often occur at the cement-trabeculae interface. Therefore, this study focused solely on investigating the micromechanics of the cement-trabeculae interface without considering the implant.

The stress discrepancy could reach up to 19% when using the conventional lab-prepared or cadaveric cement–bone interface specimen, where a micro- or macro-gap between the bone and cement is inevitable^26^. A significant advantage of our model is the complete compaction (without any gap) between the bone and cement components, which was achieved through precise Boolean logic operations during the tetrahedral-element meshing stage. This meticulous control over artificial interference ensures that the results can be considered as optimal theoretical values. Another advantage is the convenient process of FE model development, where only micro-CT images of trabecular samples are required. These images can be obtained from discarded bone samples through surgical procedures (as proposed in this study), high-resolution peripheral quantitative CT scans on living bodies^34^, or experimental scans from animal samples. By eliminating the need for lab-preparation or pre-processing involving a mixture of cadaveric bone and cement, significant reductions in operation time can be achieved. In this study, the micromotion amplitude of the interdigitated bone in all models was found to be less than 5 um while the von Mises stress exhibited significant fluctuations due to varying volume fractions; however, all values remained below 15 MPa. Previous investigations involving cadaveric bone and FE analysis have consistently reported micromovement amplitudes ranging from 0 to 5 μm and von Mises stresses within a similar range^20–22,28^. The magnitudes of micromotion or stress observed in our study align with those documented in previous research, thus validating the accuracy of our model.

We initially examined the impact of contact properties between bone and cement. Some variations were observed in micromotion and stress near the cement border when comparing friction (with a coefficient of 0.3), frictionless, and bond contacts (Fig. 4). However, these differences were found to be insignificant, aligning with a previous study^26^, indicating that the interdigitation of cement and trabecular bones predominantly governs the micromechanics at the PFNA cement interface. A prior investigation revealed that the influence of frictional contact is more pronounced during tension or shearing, where the interface exhibits greater compliance. The larger effect of using different contact properties at the cement–bone interface may be due to their cyclic loading mode as well as differences in the level of model detail, such as cube size and ID^30,35^. Reaming results in fewer trabeculae remaining in the femoral marrow cavity during cemented total hip replacement. Studies revealed that the presence of interfacial grooves, rather than interdigitation, plays a crucial role in promoting bonding, and enhancing the longevity of cemented hip prosthesis^36,37^.

A study investigating the alignment of femoral head cement-trabeculae composites with the main compressive trabecular direction revealed that at low BV/TV, the properties of the composite are predominantly influenced by cement, whereas at high BV/TV, the contribution of trabecular bone becomes more apparent, indicating a positive correlation between BV/TV and in-axis modulus^27^. In our study, we did not observe a significant correlation (R^2^ = 0.02) between the BV/TV and micromotion in the augmented trabeculae near the cement border; however, there was a significant correlation (R^2^ = 0.70) between stress and BV/TV. The observed micromotion trend contradicts previous findings, which reported higher micromotion at the contact interface for specimens with a lower BV/TV ratio^28^. This could be attributed to the following factors: (1) The trabecular specimens in the previous study were randomly obtained from the proximal tibia, which differs from our femoral head trabeculae in terms of their structural alignment with the main trabecular direction. It is well-known that site-specific differences in bone structure significantly influence the material properties of human trabecular bone^38,39^; (2) Random error may have occurred due to a small sample size (n = 4); and (3) The loading and boundary directions were completely reversed. However, the most proximal loading bone exhibited a moderate correlation (R^2^ = 0.61) between BV/TV and micromotion, as well as a significant correlation (R^2^ = 0.93) between BV/TV and stress. It is expected that the microstructure (i.e., BV/TV) would influence micromotion or stress in the loading bone; however, it is not anticipated that BV/TV would significantly impact micromotion or stress in the interdigitated bone. These asymmetrical findings suggest that cement augmentation modifies the mechanical behavior of bone-cement composites, potentially due to constraints on micromotion.

The influence of micromotion constraint was more pronounced in the loading and interdigitated bone under pre- and post-augmentation conditions.

When a thicker cement mantle was present, the most distal bone was completely encapsulated in the cement. In clinic, the optimal dosage of bone cement injection remains a subject of debate: an insufficient dose fails to enhance stability, while an excessive dose may give rise to various complications associated with bone cement (such as thermal injury, femoral head necrosis, delayed fracture healing, etc.). Studies have demonstrated that a dosage of 4.2 mL of bone cement is deemed appropriate and has been clinically established as the standard injection dose for bone cement augmentation. Furthermore, biomechanical analysis has indicated that the utilization of 4.2 mL of bone cement can effectively enhance the stability of intramedullary nails. Importantly, it should be noted that the elevation in temperature and pressure within the femoral head resulting from the application of 4.2 mL cement does not lead to femoral head necrosis^40–43^. In this study, we devised various IDs to simulate a clinical scenario involving the application of different quantities of bone cement. It is evident that as the amount of bone cement increases (i.e., with larger ID), there is a corresponding decrease in displacement and deformation of the interdigitated bones, indicating greater stability in the trabecular structure. These findings were corroborated through biomechanical test and a long-term prospective multicentre trial^14,44^.

Compared to pre-augmentation, the reduction curves of interdigitated bone micromotion exhibited a slight slope as the ID increased, while the stress reduction curves maintained a consistent slope similar to that observed before augmentation (Fig. 8). This implies that cement augmentation has a greater impact on reducing micromotion compared to stress reduction. Furthermore, there was a significant difference in micromotion between the loading bone before and after augmentation, whereas the disparity in stress appeared to be minimal (Fig. 9). The stress distribution in the loading bone is not significantly altered by cement augmentation. The asymmetrical effect observed after cement augmentation may be attributed to the unchanged trabecular structure and loading transmission route caused by the presence of a cement mantle, which restricts horizontal trabecular displacement.

The results presented in Fig. 11 demonstrate pronounced gradients of displacement and stress reduction following bone cement augmentation, particularly in cases with lower BV/TV values. This observation indicates the remarkable efficacy of bone cement augmentation for individuals with decreased bone mass^11^. Considering the macro biomechanical aspect, bone cement exhibits exceptional efficiency in managing unstable intertrochanteric fractures by generating substantial displacement and stress reduction gradients, and significantly enhancing resistance against cut-out complications^12^.

While bone cement provides initial stable fixation, it also leads to strain shielding of the trabeculae that interdigitate with the cement^28,45^. This strain-shielding effect may result in compromised interlocking and long-term aseptic loosening between the cement and trabeculae *in vivo*^20,33,46^. To our knowledge, there have been no reported cases of aseptic loosening of cement-augmented PFNA, possibly due to concerns regarding cement-related complications and its limited utilization owing to the requirement for a specially designed blade^8,9^. Considering the micromotion-constraint effect observed in our study, future reports of aseptic loosening might emerge as cement-augmented PFNA becomes more commonplace.

## Limitations

By incorporating cement directly into the model, an optimal representation of the cement-trabecular interface can be achieved. However, it should be noted that this technique is most suitable for the initial stage of bone cement injection and when there are no gaps present within the trabecular space after complete filling with cement. In fact, varying degrees of osteolysis and the formation of gaps at the cement-trabecular interface can be observed, potentially leading to an overestimation of the actual outcomes based on our model's numerical results. Due to the limited number of samples in our study, a comprehensive determination of the influence of morphological characteristics on interface properties could not be achieved. Analysing additional samples would provide more information regarding the impact of bone morphology and cement ID. The femoral head cement-bone composite primarily withstands compressive forces, although shear forces and/or a combination of shear and compression forces may be anticipated depending on the specific location within the interface in the femoral head. For this study, only compressive loads were considered as they represent the primary load pattern. Nevertheless, since the PFNA blade effectively compacts trabecular tissue in the femoral head^47,48^, additional models of cement-bone interfaces need to be developed to elucidate the impact of cement-augmented PFNA on compacted trabecular tissue.

## Conclusion

User-friendly, universal, and accurate cement-trabeculae interface FE models were developed to investigate the micromechanics of cement-augmented PFNA. Our findings indicate that: (1) the cement and trabecular bone interlock with each other; (2) micromotion and stress decrease as the ID increases. Stress at the proximal interdigitated bone is influenced by bone volume fraction (BV/TV), while both micromotion and stress at the most proximal loading region show dependence on BV/TV; (3) comparing the post- and pre-augmentation situations, micromotion reduction in the interdigitated bone is superior to stress reduction, especially in the regions near the cement border. Cementation significantly reduces micromotion in the loading bone but only marginally reduces stress. In summary, our data suggest that cement augmentation primarily controls micromotion rather than stress. This highlights the potential effectiveness of cement-augmented PFNA for controlling implant stability through minimizing micromotions.

## Data Availability

Data is provided within the manuscript.
